# Prevalence and Medical Resource of Patients with Diabetic Foot Ulcer: A Nationwide Population-Based Retrospective Cohort Study for 2001–2015 in Taiwan

**DOI:** 10.3390/ijerph18041891

**Published:** 2021-02-16

**Authors:** Chia-Hui Tai, Tsung-Cheng Hsieh, Ru-Ping Lee, Shu-Fen Lo

**Affiliations:** 1 Department of Nursing, Hualien Tzu Chi Hospital, Buddhist Tzu Chi Medical Foundation, Hualien 970473, Taiwan; 2 Institute of Medical Sciences, Tzu Chi University, Hualien 970374, Taiwan; tchsieh@gms.tcu.edu.tw (T.-C.H.); fish@mail.tcu.edu.tw (R.-P.L.)

**Keywords:** diabetic foot ulcer, DFU, medical resource, prevalence

## Abstract

Diabetic foot ulcer (DFU) is one of the common complications of diabetes. DFU can cause a huge medical and financial burden due to infections, compromise the quality of life, and increase the mortality rate in patients. However, the consumption of medical resources for DFU is rarely mentioned. A retrospective cohort study was performed. Data were obtained from the National Health Insurance Research Database of Taiwan, and the prevalence and medical utilization data for DFU in 2001–2015 were extracted, followed by the analysis for high-risk populations. Between 2001 and 2015, there were 7511 new DFU patients. A higher proportion in these patients was male, elderly with a low education level, and low income. Between 2001 and 2015, the prevalence of DFU was 0.5–0.8%, and the number of DFU patients showed stable growth. Every year, 12.6–19.3% and 1.2–7.0% of patients underwent debridement and amputation, respectively. The hospitalization fees increased year on year. Our study showed that the DFU prevalence increased year on year, and the DFU medical expenditure increased. DFU tends to occur in males, patients with low socioeconomic status, low education level, those with multiple comorbidities, and old age. Therefore, DFU care and prevention require the entire healthcare system to jointly formulate a prevention plan.

## 1. Introduction

Diabetes is a chronic metabolic disease, 90–95% of diabetics have type 2 diabetes [1]. According to a survey by the World Health Organization (WHO), there are 422 million diabetics in the world and 1.6 million deaths every year are directly caused by diabetes [2]. Diabetic complications, such as retinopathy, peripheral vasculopathy, neuropathy, and nephropathy, can cause vision loss, amputation, infection, renal failure, nerve damage, and may even be responsible for cerebral and myocardial infarction and result in death [2]. Diabetes and its complications may increase direct healthcare expenditures and considerably impact the patient’s daily life, family, and social economy. Various dermatological conditions, such as ulcers or psoriasis have been linked to diabetes [3]. Diabetic foot ulcer (DFU) occurs in types 1 and 2 diabetes mellitus and is a common complication of diabetes, i.e., lower limb lesions caused by poor long-term glycemic control [4]. The primary cause of DFU includes abnormalities in foot structure, sensation, movement, and circulation in patients, all of which are caused by diabetic neuropathy and peripheral artery disease. Note that these wounds do not easily heal in these patients [5,6]. The lifetime incidence of foot ulcer in diabetics is 25% [6]. Compared with other diabetic complications, DFU has a higher hospitalization rate, incidence, and mortality rate, and persistent worsening will cause DFU to progress to ulceration or necrosis or even lead to amputation in serious cases [5]. The amputation rate in diabetics is 10–30 times more than that in non-diabetics and account for 69–80% of non-traumatic amputations [6,7]. The risk factors of DFU include age, male, peripheral neuropathy, peripheral vasculopathy, smoking, trauma, history of diabetes for >10 years, and glycated hemoglobin of >7.5%, all of which positively correlate with DFU [8]. The medical expenditure for DFU is extremely high as it increases the length of hospitalization and the number of emergency and outpatient visits. Moreover, DFU increases the medical cost by 28,593 USD every year [9]. In the United Kingdom, the annual medical insurance expenditure for DFU and amputation in 2014–2015 is estimated to be GBP 837–962 million and accounts for 0.8–0.9% of the National Health Service budget. Note that >90% of expenditure is related to long-term and severe ulcers, and the length of hospitalization increases by 8.04 days in DFU patients compared with diabetics without ulcers [10]. This study aims to use the epidemiological characteristics of DFU patients described in Taiwan’s National Health Insurance Research Database (NHIRD), namely, outpatient consultation and inpatient records, to understand the consultation status and geographical distribution to examine DFU prevalence, risk factors, and medical utilization status.

## 2. Materials and Methods

### 2.1. Data Sources

In this study, NHIRD data were used for the analysis. The national health insurance (NHI) system was set up by the Taiwanese government in 1995 and is mandatory for all Taiwanese citizens. The NHI coverage of 99% and the database can be used to monitor and evaluate medical services [11]. In this study, we analyzed the occurrence trends and medical resource consumption for DFU. The study data were from a cohort of 2 million sampled people and included all persons’ consultation and declaration data from 2000 to 2015. An encrypted code was used for linking. This study was approved by the Research Ethics Committee of Hualien Tzu Chi Hospital (IRB107-91-C). In this study, the diagnosis code for diabetes (250) in the International Classification of diseases, 9th Revision with Clinical Modification (ICD-9CM code) was used to screen and diagnose patients with diabetes. The diagnostic criteria for diabetes are as follows: A diabetes diagnosis in outpatient (at least three times) and inpatient (at least one time) records.

### 2.2. Definition of Diabetes Foot Ulcer and Medical Resource

The screening method of Lin et al. [12] was used as a reference for DFU diagnosis. Patients with DFU are diabetics with a diagnosis code of foot ulcers (ICD-9CM code, 707.06, 707.07, 707.1, 892, and 893). Patients who were diagnosed with DFU before 31 December 2000 and <20 years were excluded to confirm the trend of new DFU diagnosis, time of DFU onset, and medical utilization between 2001 and 2015. Furthermore, we examined patient characteristics (such as sex, age, major illness, the location of medical consultation, economic status), Charlson comorbidity index (CCI), type of inpatient department, length of hospitalization, surgery status, and medical resource consumption, and their correlations. The medical resource consumption was the sum of a total length of hospitalization due to DFU. The annual medical expenditure for every DFU patient was obtained from the NHI, and all the fees were converted to USD (using the USD exchange rate, 30 Taiwanese dollars to the USD dollar, in 2020).

### 2.3. Statistical Analysis

Descriptive statistics and inferential statistics were primarily used for the analysis. For descriptive statistics, the number of subjects and percentage were used to describe the categorical variables of DFU patients (sex, location of medical consultation, type of consultation department, surgery status, the number of DFU cases every year, and annual incidence) and distribution. The mean and standard deviation or median and IQR were used to describe the distribution of continuous variables (age, length of hospitalization, and medical expenditure). SPSS 18.0 (IBM, Armonk, NY, USA) and SAS 9.4 (SAS Institute Inc., Cary, NC, USA) were used for statistical analysis. Excel (Microsoft, Redmond, Washington, DC, USA) was used to generate graphs and tables.

## 3. Results

### 3.1. General Characteristics of the Participants

The number of diabetics increased 2.6 times from 61,593 in 2001 to 162,873 in 2015. The prevalence of diabetes increased from 4.75% in 2001 to 10.15% in 2015. The disease diagnosis code was used to screen outpatient and inpatient information. After excluding repeated patients and patients who developed DFU before 2000, there were 7511 new DFU patients during 2001–2015. The mean age was 63.2 ± 13.5 years, duration of DFU (time from the DFU diagnosis to death or end of observation) was 6.5 ± 4.5 years (median: 6.2 years, IQR = 2.5–10.2), and 33.8% (*n* = 2538) DFU patients died during the observation period. There were slightly more males (55.8%) than females (44.2%). Subjects (59.3%) have an education level below the elementary school, and 66.5% of subjects were married. Most subjects (33.4%) were first diagnosed with DFU in the surgery department, followed by the internal medicine (21.0%) and plastic surgery (20.0%) departments. The CCI was 2.5 ± 1.8. Most patients (73.7%) had comorbidity of diabetes 1 year before the DFU diagnosis, followed by hypertension (55.4%), coronary heart disease (24.6%), ulcerative disease (17.8%), and cerebrovascular disease (17.1%). Table 1 shows the patient characteristics.

### 3.2. DFU Prevalence

The prevalence of DFU during 2001–2015 was between 0.52% and 0.76% (Table 2), and the DFU prevalence was higher in 2005 (0.76%). There were differences in the prevalence between sexes and age groups. There were more male DFU patients than females, and the prevalence of DFU in patients aged 65 years and above was higher than the other age groups.

### 3.3. Medical Expenditure Due to DFU

Table 1 shows the consultation status and medical expenditure for 7511 DFU patients from 2001 to 2015. The median number of outpatient visits was 170 (IQR: 71–325), the median number of hospitalizations was 4 (IQR: 2–9), and the length of hospitalization was 42 days (IQR: 15–99). The median number of outpatient visits due to DFU was 2 (IQR: 1–6), the median number of hospitalizations due to DFU was 1 (IQR: 1–2), and the length of hospitalizations due DFU was 13 days (IQR: 7–26). Moreover, 20–30% of hospitalization fees were caused by DFU. In addition, 18.6% of DFU patients underwent amputation, and 37.4% of DFU patients underwent debridement. Table 3 shows the annual medical expenditure for DFU during 2001–2015. The data show that the number of outpatient visits, number of hospitalizations, and outpatient medical fees from 2001 to 2015 was similar, but hospitalization fees increased year by year at 1.8–2.1 times during the 15-year period. Every year, 12.6–19.3% and 1.2–7.0% of patients underwent debridement and amputation, respectively.

## 4. Discussion

Our study aims to understand the epidemiological trends and medical expenditure due to DFU from 2001 to 2015. In this study, we observed that the prevalence of diabetes increased year by year. From 2001 to 2015, the number of diabetes and DFU patients increased 2.6 times each. The prevalence of DFU was 0.5–0.8% and slightly decreased in 2012. Note that additional studies may be required to examine if this decrease is caused by the expansion of the Diabetes Shared Care Network. The Diabetes Shared Care Network was initiated in I-Lan County in 1996 to provide education and long-term follow-up for registered patients with diabetes by multidisciplinary diabetes care teams. This program was extended to include patients across Taiwan in 2002. The program has been promoted for a long time with the aim of improving blood glucose levels and reducing the incidence of complications in patients with diabetes [13]. The results in Table 2 show that the DFU prevalence is higher in males, older subjects, subjects with many comorbidities, lower financial status, and lower education level, which are similar to those of Lin et al. [12]. Our study included prevalence, outpatient and consultation status, and medical expenditure, which enables us to comprehensively understand the medical utilization for DFU.

Between 2001 and 2015, there were 7511 newly diagnosed DFU patients. Most patients suffered from chronic comorbid diseases with high medical requirements. Among these patients, inpatient fees due to the DFU account for >20–30% of total inpatient expenditure in these patients. The occurrence of DFU indicates that the medical expenditure is increased. In this study, we reported that most DFU patients have a low financial status, and the fees for medical care in these patients may be higher than their income, which may increase the financial burden in these patients.

In this study, we reported that the amputation rate of DFU patients in 2001–2015 was 1.2–7.0%, which was similar to the study of Lin et al. and Geiss et al. in the US [6,11], but the amputation rate in this study was higher. Lin et al. and Geiss et al. mainly analyzed DM patients, and their amputation rates were 2.1–2.9% and 3.1–5.4%, respectively. In this study, the subjects were DFU patients, and the amputation rate was 1.2–7.0%, which shows that DFU is associated with a high amputation rate [5,6].

Our study examined the debridement in DFU patients, and the ratio of DFU patients who undergo debridement every year was 12.6–19.3%. However, this study was restricted to information obtained from diagnosis codes, and there was a lack of information on DFU severity, number of wounds, duration, lab data, and the success of surgery or debridement. This study only considered data from a national database; thus, the actual prevalence of these conditions may be higher. In the future, medical records in medical institutions may be combined for further analysis and formulation of care strategies.

## 5. Conclusions

This study aims to understand the prevalence trends and medical utilization of DFU. The results showed that the diabetes prevalence increased year on year in Taiwan, and the number of DFU patients and medical expenditure increased. This phenomenon may gradually worsen as the number of diabetics and comorbidities increases, thereby causing public health and economic problems. Therefore, the prevention and appropriate treatment for DFU require the entire healthcare system to formulate a DFU care plan together. Our study quantified the epidemiological trend for DFU, which facilitates the formulation of comprehensive prevention policies and care guidelines.

## Figures and Tables

**Table 1:** Characteristics of diabetic foot ulcer (DFU) patients (*N* = 7511).

Variable	*n*	Percentage
Age		
20–45	641	8.5
45–65	3329	44.3
≥65	3541	47.2
CCI		
0	927	12.3
1	1504	20.0
2	1883	25.1
≥3	3197	42.6
Gender		
Male	4194	55.8
Female	3317	44.2
Education level		
Below Elementary school	4455	59.3
Middle school	1289	17.2
High school	1223	16.3
College or above	544	7.2
Location of residence		
North	2548	33.9
Central	2820	37.5
South	1636	21.8
East	507	6.8
Marital status		
unmarried	561	7.5
married	4995	66.5
divorced/widowed	1955	26.0
Socioeconomic status		
Low	3497	46.5
Moderate	3702	49.3
High	312	4.2
Amputation	1400	18.6
Debridement	2806	37.4
Comorbidity (1 year before the DFU diagnosis)		
Diabetes	5335	71.0
Hypertension	4158	52.6
Coronary heart disease	1851	24.6
Ulcer disease	1340	17.8
Cerebrovascular disease	1282	17.1
Chronic pulmonary disease	1220	16.2
Moderate or severe renal disease	1128	15.0
Mild liver disease	703	9.4
Peripheral vascular disease	679	9.0
Congestive heart failure	616	8.2
Dementia	285	3.8
Hemiplegia	106	1.4
**Variable**	**Median**	**IQR**
Overall consultation status		
Number of outpatient visits	170	71–325
Outpatient fees (USD)	8234.59	3073.52–17,854.97
Number of hospitalizations	4	2–9
Hospitalization fees (USD)	257,897	3166.34–20,807.52
Length of hospitalization (days)	42	15–99
DFU consultation status		
Number of outpatient visits	2	1–6
Outpatient fees (USD)	52.62	24.48–144.14.
Number of hospitalizations	1	1–2
Hospitalization fees (USD)	1954.72	817.86–4372.48.
Length of hospitalization (days)	13	7–26

CCI: Charlson comorbidity index; DFU: Diabetic foot ulcer; USD: United States Dollar.

**Table 2:** DFU prevalence during 2001–2015.

Variable	2001	2002	2003	2004	2005	2006	2007	2008	2009	2010	2011	2012	2013	2014	2015
DM patient (N)	61,593	67,038	71,429	80,325	86,465	93,261	102,017	111,391	122,841	129,375	136,305	143,912	151,313	157,103	162,873
DM prevalence (%)	4.75	5.04	5.24	5.77	6.09	6.46	6.95	7.45	8.08	8.43	8.81	9.22	9.60	9.87	10.15
DFU prevalence (%)															
Overall	0.53	0.64	0.63	0.73	0.76	0.70	0.69	0.69	0.68	0.68	0.69	0.61	0.58	0.60	0.52
Gender															
Male	0.56	0.72	0.72	0.80	0.85	0.76	0.78	0.80	0.79	0.77	0.82	0.71	0.68	0.70	0.58
Female	0.50	0.57	0.55	0.66	0.67	0.65	0.60	0.59	0.58	0.58	0.55	0.50	0.47	0.49	0.46
Age															
20–50	0.37	0.57	0.47	0.57	0.59	0.49	0.56	0.50	0.53	0.51	0.50	0.47	0.51	0.50	0.43
50–65	0.53	0.65	0.71	0.76	0.73	0.69	0.65	0.67	0.62	0.63	0.62	0.53	0.56	0.53	0.43
≥65	0.70	0.70	0.68	0.81	0.90	0.85	0.80	0.82	0.83	0.80	0.84	0.75	0.62	0.70	0.65
Socioeconomic status															
Low	0.56	0.64	0.63	0.66	0.74	0.71	0.74	0.75	0.69	0.73	0.74	0.64	0.64	0.63	0.60
Middle	0.55	0.69	0.68	0.84	0.83	0.75	0.69	0.68	0.72	0.69	0.69	0.63	0.58	0.61	0.50
High	0.19	0.27	0.39	0.41	0.40	0.34	0.32	0.41	0.39	0.30	0.38	0.31	0.25	0.32	0.27
Location of residence															
North	0.36	0.44	0.47	0.56	0.50	0.55	0.54	0.57	0.53	0.57	0.53	0.50	0.49	0.50	0.45
Central	0.57	0.72	0.68	0.76	0.88	0.74	0.76	0.75	0.72	0.72	0.73	0.63	0.61	0.63	0.57
South	0.76	0.91	0.90	1.03	1.07	0.91	0.89	0.79	0.87	0.81	0.86	0.79	0.67	0.69	0.58
East	0.81	0.79	0.71	0.79	0.92	0.99	0.69	0.96	1.05	0.81	1.09	0.73	0.70	0.83	0.58
Marital status															
unmarried	0.51	0.70	0.66	0.67	0.86	0.76	0.78	0.81	0.74	0.83	0.86	0.67	0.78	0.62	0.58
married	0.50	0.61	0.58	0.70	0.72	0.69	0.64	0.64	0.61	0.62	0.63	0.57	0.52	0.58	0.47
divorced/widowed	0.69	0.78	0.94	0.90	0.86	0.73	0.82	0.83	0.91	0.82	0.84	0.73	0.68	0.62	0.69
Education level															
Below Elementary school	0.66	0.81	0.84	0.92	0.96	0.90	0.80	0.84	0.87	0.82	0.84	0.75	0.70	0.75	0.68
Middle school	0.53	0.66	0.56	0.73	0.74	0.68	0.80	0.71	0.74	0.75	0.80	0.69	0.62	0.62	0.56
High school	0.37	0.47	0.32	0.49	0.51	0.50	0.58	0.59	0.48	0.53	0.54	0.50	0.50	0.53	0.43
College or above	0.24	0.21	0.34	0.36	0.36	0.31	0.30	0.32	0.28	0.34	0.32	0.26	0.30	0.30	0.23

DM: Diabetes Mellitus; DFU: Diabetic foot ulcer.

**Table 3:** Medical expenditure for DFU during 2001–2015.

Variable	2001	2002	2003	2004	2005	2006	2007	2008	2009	2010	2011	2012	2013	2014	2015
Patients with DFU	325	429	453	585	655	657	700	771	840	875	935	875	872	937	849
Debridement (%)	16.6	15.4	16.1	19.0	15.7	15.7	17.1	17.6	19.3	19.1	16.9	12.6	14.9	16.1	14.1
Amputation (%)	1.2	2.8	3.8	4.1	5.0	5.6	5.4	6.6	5.6	6.1	7.0	4.8	4.9	5.1	4.7
Number of outpatient visits *	2 (1–5)	2 (1–4)	2 (1–5)	2 (1–6)	2 (1–5)	2 (1–5)	2 (1–5)	2 (1–6)	2 (1–4)	2 (1–5)	2 (1–6)	2 (1–6)	2 (1–5)	2 (1–5)	2 (1–5)
Outpatient fees (USD) *	48.59 (21.21–127.10)	36.03 (21.10–105.59)	47.79 (21.28–141.03)	57.07 (23.76–175.55)	47.14 (24.38–119.48)	44.76 (22.97–120.10)	50.45 (22.21–131.38)	53.79 (23.34–141.86)	50.62 (22.53–122.14)	49.97 (23.10–128.93)	51.38 (25.10–146.03)	57.59 (25.66–164.17)	51.69 (25.07–138.79)	49.59 (23.59–137.17)	49.21 (25.03–139.03)
Number of hospitalizations *	1 (1–1)	1 (1–1)	1 (1–1)	1 (1–1)	1(1–1)	1 (1–1)	1 (1–1)	1 (1–1)	1 (1–1)	1 (1–1)	1 (1–1)	1 (1–1)	1 (1–1)	1 (1–1)	1 (1–1)
Hospitalization fees (USD) *	925.90 (486.41–1808.10)	1176.91 (537.45–2439.83)	1259.03 (565.07–2726.69)	1340.90 (701.72–2917.59)	1609.86 (701.72–3693.03)	1397.55 (718.45–2799.31)	1457.59 (728.48–3797.52)	1606.10 (781.00–3395.59)	1806.52 (771.00–3512.07)	1668.86 (810.21–3398.07)	1730.41 (847.38–3424.97)	1523.79 (871.86–3016.83)	1815.79 (887.52–4084.45)	1982.34 (903.83–3795.62)	1949.24 (905.97–3873.03)
Length of hospitalization (days) *	8 (5–18)	10 (5–24)	11 (6–22)	11 (7–24)	11 (6–21)	11 (6–20)	10 (5–24)	11 (6–22)	12 (6–22)	10 (6–20)	11 (6–20)	10 (6–18)	11 (6–20)	11 (6–21)	10 (6–19)

DFU: Diabetic foot ulcer; USD: United States Dollar; * Median (IQR).

## Data Availability

Data sharing is not applicable.
